# Concordance and predictive value of two adverse drug event data sets

**DOI:** 10.1186/1472-6947-14-74

**Published:** 2014-08-22

**Authors:** Aurel Cami, Ben Y Reis

**Affiliations:** 1 Division of Emergency Medicine, Boston Children’s Hospital, 1 Autumn Street, 5th Floor, Boston, MA 02215, USA; 2 Department of Pediatrics, Harvard Medical School, Boston, MA, USA

**Keywords:** Adverse drug events, Prediction, Concordance

## Abstract

**Background:**

Accurate prediction of adverse drug events (ADEs) is an important means of controlling and reducing drug-related morbidity and mortality. Since no single “gold standard” ADE data set exists, a range of different drug safety data sets are currently used for developing ADE prediction models. There is a critical need to assess the degree of concordance between these various ADE data sets and to validate ADE prediction models against multiple reference standards.

**Methods:**

We systematically evaluated the concordance of two widely used ADE data sets – Lexi-comp from 2010 and SIDER from 2012. The strength of the association between ADE (drug) counts in Lexi-comp and SIDER was assessed using Spearman rank correlation, while the differences between the two data sets were characterized in terms of drug categories, ADE categories and ADE frequencies. We also performed a comparative validation of the Predictive Pharmacosafety Networks (PPN) model using both ADE data sets. The predictive power of PPN using each of the two validation sets was assessed using the area under Receiver Operating Characteristic curve (AUROC).

**Results:**

The correlations between the counts of ADEs and drugs in the two data sets were 0.84 (95% CI: 0.82-0.86) and 0.92 (95% CI: 0.91-0.93), respectively. Relative to an earlier snapshot of Lexi-comp from 2005, Lexi-comp 2010 and SIDER 2012 introduced a mean of 1,973 and 4,810 new drug-ADE associations per year, respectively. The difference between these two data sets was most pronounced for Nervous System and Anti-infective drugs, Gastrointestinal and Nervous System ADEs, and postmarketing ADEs. A minor difference of 1.1% was found in the AUROC of PPN when SIDER 2012 was used for validation instead of Lexi-comp 2010.

**Conclusions:**

In conclusion, the ADE and drug counts in Lexi-comp and SIDER data sets were highly correlated and the choice of validation set did not greatly affect the overall prediction performance of PPN. Our results also suggest that it is important to be aware of the differences that exist among ADE data sets, especially in modeling applications focused on specific drug and ADE categories.

## Background

Predictive modeling of adverse drug events (ADEs) is attracting growing interest. The high ADE-related costs in the US have been known for many years [1,2], and recent studies conducted in other developed countries provide further motivation for the importance of this problem worldwide [3-5]. To address these large and growing ADE-related economic and public health concerns, a wide array of ADE identification and prevention methods have been implemented. These include early-stage drug toxicity prediction and testing [6-8], clinical trials for evaluating a drug’s safety profile, and post-market surveillance methods for detecting abnormally high ADE rates [9,10]. Still, many types of ADEs can go undetected for years after a drug has been on the market [11,12], necessitating constant additions of label warnings and, in extreme cases, drug withdrawals [13,14]. At the same time, toxicity and clinical safety concerns remain lead causes of the high attrition rates in the drug development process [15], as the cost of bringing New Molecular Entities (NMEs) to the market continues to increase [16]. All these factors have spurred a noticeable expansion in research on adverse event prediction – research that critically relies on good data. The emerging field of system pharmacology is being increasingly recognized as a promising new approach for predicting ADEs [17]. System pharmacological approaches typically rely on the integration of various diverse types of data, such as chemical, biological and taxonomic, followed by the application of quantitative models to extract information from these data. Often times, the data are represented and integrated through network models [18,19]. In recent years, a number of system pharmacology predictive models for ADEs have been proposed [20-27].

While all these predictive approaches rely critically on “known” drug-ADE associations, there is currently no “gold standard” source for drug safety data. As a result, several different ADE data sets have been used to develop predictive pharmacological models. Often, the drug-ADE associations listed in these data sets are primarily extracted from drug package inserts. This is the case, for instance, with SIDER – a widely used public database, and Lexi-comp – a widely used commercial database. Alternatively, the listed drug-ADE pairs may be extracted from post-marketing databases, such as FAERS (formerly AERS; http://www.fda.gov/Drugs). Thus, the drug safety data used to train the above models may contain drug-ADE associations supported by strong evidence (e.g. associations for whom a causal link between the drug and ADE has rigorously been established) as well as associations supported by weaker evidence (e.g. associations based solely on post-marketing reports).

Recent work has highlighted various types of inconsistencies in the reporting of drug safety information, including discrepancies among the reports for bio-equivalent drugs, or reports used in different countries [28-30]. However, systematic comparisons of the major safety data sets used in system pharmacological models and assessment of the impact of data-set choice on prediction performance are lacking. In this paper, we systematically compare the Lexi-comp and SIDER ADE data sets. While the choice of an ADE data set can also have diverse clinical and economic consequences, we focus on its implications for predictive models. As a case study, we use the Predictive Pharmacosafety Networks (PPNs) model.

## Methods

### Framework overview

**Figure 1:**
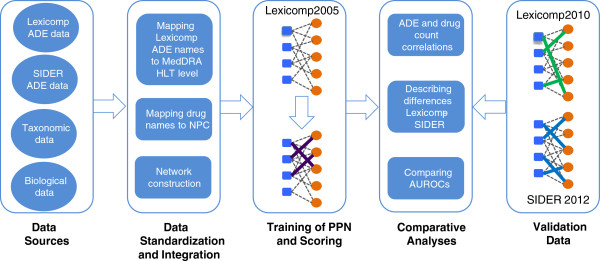
**Overview of the study framework.** First, data were integrated from multiple sources, including data on drug-ADE associations from two different sources (Lexi-comp and SIDER), drug and ADE taxonomies, and intrinsic drug properties. Next, a number of steps to standardize and integrate these data were carried out. The strength of the association between the counts of ADEs and drugs in Lexi-comp and SIDER data sets and the differences between these two data sets were assessed. Finally, a PPN model was trained using a 2005 version of Lexi-comp and validated using both Lexi-comp 2010 and SIDER 2012 as reference standards.

Figure 1 shows an overview of the study framework. First, we integrated data from multiple sources, including data on drug-ADE associations from two snapshots of Lexi-comp and one snapshot of SIDER, drug and ADE taxonomies, and intrinsic drug properties. Next, we carried out a number of steps to standardize and integrate these data, including mapping the Lexi-comp and SIDER ADE names to MedDRA High Level Terms (HLTs), standardizing the drug names in Lexi-comp and SIDER, and constructing bi-partite network representations of the drug-ADE associations. Next, we assessed the strength of the association between the counts of ADEs (per drug) and drugs (per ADE) in Lexi-comp and SIDER data sets. We also identified the difference between the two data sets and characterized it in terms of drug categories, ADE categories and ADE frequencies. Finally, we trained a PPN model using a 2005 version of Lexi-comp data and validated the prediction performance of PPN using both Lexi-comp 2010 and SIDER 2012 as reference standards.

### Data description

The NCGC Pharmaceutical Collection (NPC) resource [31] was used to identify different forms of drug names that refer to a common “active pharmaceutical ingredient”. The Medical Dictionary for Regulatory Activities (MedDRA) standard (http://www.meddra.org) was used to code ADE names. Drug attributes were extracted from World Health Organization Anatomical Therapeutic Chemical Classification System (ATC) (http://www.whocc.no/atc), University of Alberta DrugBank (http://www.drugbank.ca), and National Center for Biotechnology Information’s Pubchem Compound (http://www.ncbi.nlm.nih.gov/pccompound).

The Lexi-comp ADE data were extracted from Lexi-Drugs® (http://www.lexi.com), a commercial database widely used in hospitals today. For this study, we had access to data on 809 Lexi-comp drugs. For each drug, we were provided with two text fields extracted, respectively, from 2005 and 2010 versions of Lexi-Drugs®. Each of these text fields integrates ADE information from drug package inserts, relevant clinical trials, case studies and post-marketing reports. We extracted the ADE names contained in each field and mapped them to MedDRA Preferred Term (PT) level. In order to compress the space of all possible drug-ADE pairs, each PT was further mapped to one or more High-Level Terms (HLT) in the MedDRA hierarchy [21]. As an example, the PT “myocardial infarction” was mapped to the following two HLTs: “ischaemic coronary artery disorders”, and “coronary necrosis and vascular insufficiency”. Since several different PTs could typically map to the same HLT, this mapping compresses the space of possible drug-ADE pairs, thereby reducing the computational time needed to train and validate the prediction model. The main trade-off of such mapping is that more complex follow-up investigations would be needed to evaluate a predicted drug-HLT pair because only a subset of the PTs mapping to the predicted HLT may actually be associated with the drug. After these pre-processing steps, the final Lexi-comp data used in our study consisted of two lists of pairs of the form (drug name, HLT name) – one list corresponding to 2005 and another to 2010.

The SIDER data used here is the most recent version of the publicly available SIDER2 database, released in October 2012. We downloaded these data from the FTP site ftp://sideeffects.embl.de/SIDER/2012-10-17/. Although earlier versions of SIDER going back to 2009 are available, SIDER2 is the first version of the database providing MedDRA-coded ADEs, making it suitable for this comparative study. SIDER integrates ADE information contained in package inserts and post-marketing reports using five main public sources: British Columbia Cancer Agency (http://www.bccancer.bc.ca), Facts@FDA (http://www.fda.gov), FDA Center for Drug Evaluation and Research (http://www.fda.gov), FDA MedWatch (http://www.fda.gov/) [32].

To enable the comparison of Lexi-comp and SIDER data, we standardized the drug names in both data sets using the NPC resource [31], and mapped SIDER ADE names from the Preferred Term level to the High-Level Terms level of MedDRA. The final pre-processed SIDER data used in our study consisted of a lists of pairs of the form (drug name, HLT name) corresponding to year 2012.

### Overview of PPNs

Predictive Pharmacosafety Networks (PPN) [21] are predictive models that exploit the overall network structure of all known drug-ADE relationships and combine it with inherent attributes of drugs and adverse events in order to predict unknown adverse events. Rather than waiting for sufficient post-marketing evidence to accumulate for a specific ADE, this predictive approach relies on leveraging contextual information from previously known drug-safety relationships, and thus has the potential to predict certain candidate ADEs earlier than they can be detected by existing pharmacovigilance methods.

Here, we provide a brief overview of the PPN model; complete details of the model, including a full specification of the data sets used to train the model, are given in Cami et al. [21]. Using logistic regression, we model the presence or absence of drug-ADE associations *Y*_*ij*_*, i* = 1,…,number of drugs, *j* = 1,…,number of ADEs, as a Bernoulli random variable and a function of three types of covariates. Network covariates depend only on the structure of the bipartite drug-ADE network. Taxonomic covariates depend on the structure of the drug-ADE network and on ATC and MedDRA codes. Intrinsic covariates depend on the structure of the drug-ADE network and on the intrinsic drug properties. Model fitting is carried out by maximum likelihood. After the model is estimated, each drug-ADE pair (*i*, *j*) not reported to be an association in the training data is scored using the predicted probabilities generated by the model.

Cami et al. [21] used the Lexi-comp 2005 ADE data to form a bi-partite network that contained 39,591 links among 809 drugs and 852 HLTS. The drug and ADE attributes described above were integrated with the nodes of this network. Twelve predictor variables were then computed and a logistic regression (LR) model was estimated. This estimated LR model achieved an area under the Receiver Operating Characteristic curve (AUROC) of 0.87 in predicting the 10,845 drug-ADE associations that were newly reported in the 2010 version of Lexi-comp.

As case studies, eight prominent drug-ADE associations discovered during the period 2006–2010 were identified by two pharmacologists. For each case study, the specificity and the positive predictive value corresponding to the score generated by PPN were computed. It was found that the specificities corresponding to the model-generated scores were consistently high, providing additional support on the utility of the model. For example, the pair (norfloxacin, tendon ruptures) achieved a specificity of 0.95, while (zonisamide, suicidal ideation) a specificity of 0.93.

### Comparative analysis

Since the Lexi-comp data available to us consisted of two snapshots from 2005 and 2010, ideally the comparison of Lexi-comp and SIDER would have been based on two SIDER snapshots from 2005 and 2010. However, the MedDRA-coded SIDER data available to us consisted of only one snapshot from 2012. Due to this restriction, we designed the comparative analysis as follows. We first identified the Lexi-comp 2010 drugs and ADEs from Cami et al. [21] study that were also included in SIDER 2012. Our goal was to assess the concordance of the sets of associations newly reported between these common drugs and ADEs in Lexi-comp 2010 and SIDER 2012, as well as the impact of data set choice on the prediction performance of PPN. In this analysis, we did not address any discordance between the sets of drug-ADE associations formed by drugs or ADEs that were included in only one of the two data sets.

In the first part of the comparative analysis, we assessed the strength of the association between ADE (or, drug) counts in Lexi-comp 2010 and SIDER 2012 by computing Spearman rank correlation. Next, we computed the difference between the Lexi-comp 2010 and SIDER 2012 data sets and characterized it in terms of drug categories, ADE categories and ADE frequencies. As described earlier, both Lexi-comp and SIDER use package inserts as the primary source of information. However, we expected that these data could differ for a number of reasons. First, SIDER 2012 may include new drug-ADE associations that were discovered after 2010 and thus could not be reported in the Lexi-comp data sets. Second, both Lexi-comp and SIDER supplement the package-inserts with other information extracted from various sources, as described earlier. Third, the mapping of ADE names to MedDRA– which, was independently implemented in the two data sets – is a non-deterministic process. There are numerous ADE names that appear in the original data sources for whom there is no exact match to MedDRA and for whom the most appropriate MedDRA code is determined either algorithmically or based on expert opinion.

In the second part of the comparative analysis, we trained a PPN model using Lexi-comp 2005 and then assessed the prediction performance of the model using Lexi-comp 2010 and SIDER 2012, respectively, as validation sets.

In the Discussion section, we explain how the data restriction mentioned earlier, i.e. the availability of only one SIDER snapshot taken at a different time point from either Lexi-comp snapshot, impacts the interpretation of our results.

## Results

### Common drugs and ADEs

Out of 809 Lexi-comp drugs used in the study by Cami et al. [21], 695 (86%) were also included in SIDER; out of 852 HLTs used in [21], 765 (90%) were also included in SIDER. In the reminder of this section, we compare the drug-ADE associations reported in Lexi-comp 2010 and SIDER 2012 between these common 695 drugs and 765 HLTs.

### Correlation between drug and ADE counts

**Figure 2:**
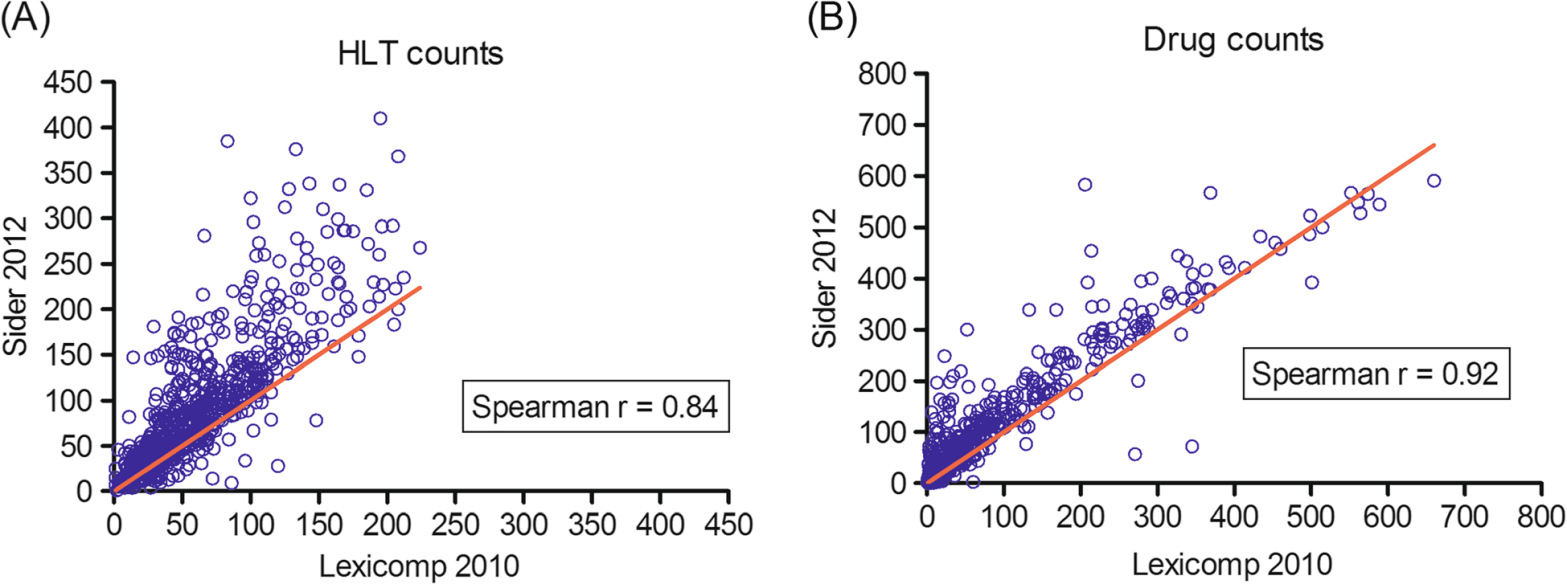
**Scatter plots of HLT and drug counts from Lexi-comp 2010 and SIDER 2012. (A)** HLT counts and **(B)** drug counts.

The Spearman correlation between the ADE counts in the Lexi-comp 2010 and SIDER 2012 data sets was 0.84 (95% CI: 0.82-0.86) (Figure 2(A)), while the Spearman correlation between the drug counts in these two data sets was 0.92 (95% CI: 0.91-0.93) (Figure 2(B)). For comparison, we also computed the Spearman correlations of ADE and drug counts between Lexi-comp 2005 and Lexi-comp 2010 data sets. For these two data sets, the correlations were, respectively, 0.87 and 0.99.

### Differences between Lexi-comp and SIDER data sets

Figure 2 indicates that drug and ADE counts generated from SIDER 2012 are generally higher than the corresponding counts from Lexi-comp 2010. In aggregate, we found that relative to the Lexi-comp 2005 data snapshot, Lexi-comp 2010 and SIDER 2012 introduced new drug-ADE associations at a mean rate of 1,973 and 4,810 per year, respectively. To better understand this difference between Lexi-comp 2010 and SIDER 2012, we computed the percentage of SIDER-only associations in each ATC top-level category, each MedDRA top-level category, and each ADE frequency group (“postmarketing”, “frequent”, “rare”, “potential”, or exact number).

We found that the drug categories with the highest percentages of SIDER-only ADEs were drugs targeting the Nervous System and Antiinfective drugs (21.9% and 11.4%, respectively, Table 1), while the ADE categories with the highest percentages of SIDER-only drugs were Nervous System Disorders and Gastrointestinal Disorders (8.9% and 8.7%, respectively, Table 2). With regards to ADE frequency classes, we found that for 63% of SIDER-only associations the frequency of ADE was missing. Of the remaining SIDER-only associations, the type having the highest percentage was “postmarketing” (46%), followed by “infrequent” (16%), “exact number” (16%), “rare” (11%), “potential” (8%), and “frequent” (3%).

**Table 1:** Number and percentage of SIDER-only associations by ATC top-level category

**ATC top-level category**	**Number (percent) of SIDER-only associations**
Nervous system	7641 (21.9%)
Antiinfectives for systemic use	3993 (11.4%)
Cardiovascular system	3894 (11.2%)
Antineoplastic and immunomodulating agents	3704 (10.6%)
Sensory organs	3672 (10.5%)
Alimentary tract and metabolism	2573 (7.4%)
Musculo-skeletal system	2300 (6.6%)
Genito-urinary system and sex hormones	2157 (6.2%)
Dermatologicals	2024 (5.8%)
Respiratory system	1378 (3.9%)
Systemic hormonal preparations (excl sex hormones)	693 (2.0%)
Blood and blood forming organs	514 (1.5%)
Various	219 (0.6%)
Antiparasitic products insecticides and repellents	146 (0.4%)

**Table 2:** Number and percentage of SIDER-only associations by MedDRA top-level category

**MedDRA top-level category**	**Number (percent) of SIDER-only associations**
Nervous system disorders	3091 (8.9%)
Gastrointestinal disorders	3038 (8.7%)
Skin and subcutaneous tissue disorders	2948 (8.4%)
General disorders	2487 (7.1%)
Respiratory, thoracic and mediastinal disorders	2309 (6.6%)
Vascular disorders	2251 (6.4%)
Psychiatric disorders	2069 (5.9%)
Musculoskeletal and connective tissue disorders	1764 (5.1%)
Eye disorders	1751 (5.0%)
Infections and infestations	1713 (4.9%)
Investigations	1477 (4.2%)
Metabolism and nutrition disorders	1417 (4.1%)
Blood and lymphatic system disorders	1287 (3.7%)
Cardiac disorders	1211 (3.5%)
Renal and urinary disorders	1116 (3.2%)
Reproductive system and breast disorders	1014 (2.9%)
Immune system disorders	784 (2.2%)
Neoplasms benign, malignant and unspecified	627 (1.8%)
Hepatobiliary disorders	614 (1.8%)
Injury, poisoning and procedural disorders	524 (1.5%)
Endocrine disorders	409 (1.2%)
Ear and labyrinth disorders	388 (1.1%)
Surgical and medical procedures	276 (0.8%)
Pregnancy, puerperium and perinatal conditions	218 (0.6%)
Congenital, familial and genetic disorders	125 (0.4%)

### Prediction performance of PPN

**Figure 3:**
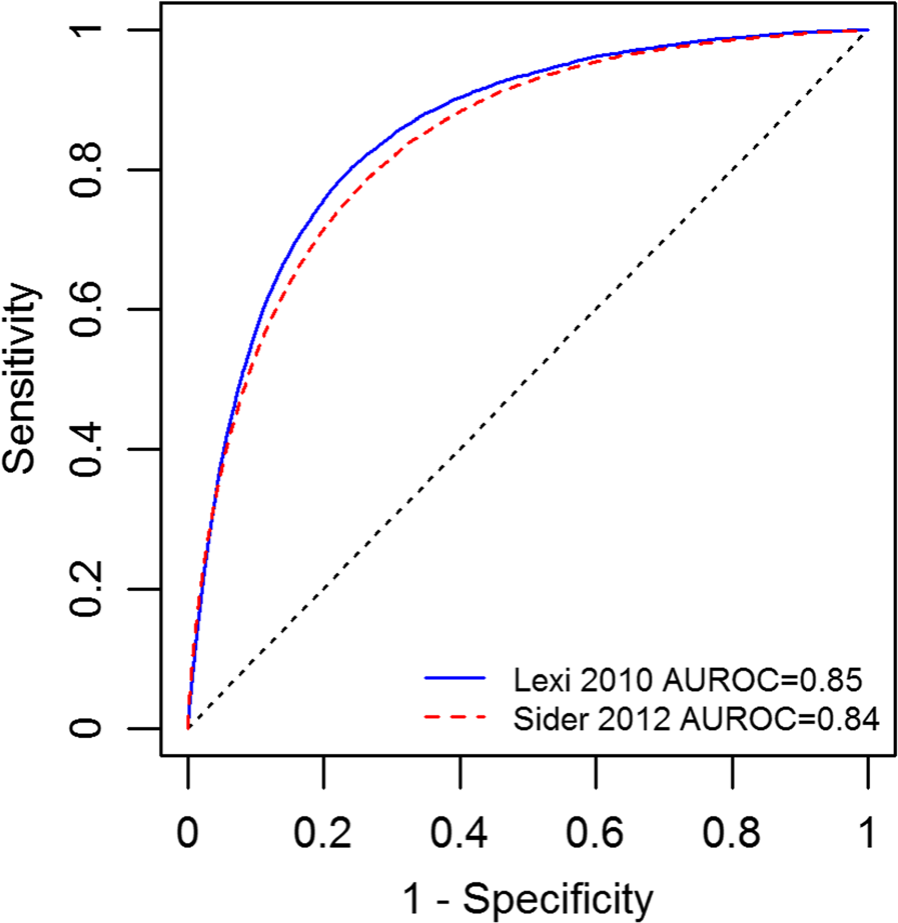
ROC curves of PPN model corresponding to the Lexi-comp 2010 and SIDER 2012 reference standards.

We compared the predictive scores generated by the PPN model trained on data from Lexi-comp 2005 with the newly introduced drug-ADE associations in Lexi-comp 2010 and SIDER 2012 data sets. We found a very minor overall change in the Area Under the Receiver Operating Characteristic curve (AUROC) of the PPN model when SIDER 2012 data were used for validation instead of Lexi-comp 2010 data: 0.84 vs. 0.85, respectively (Figure 3). Stratification of AUROC by top-level MedDRA category – i.e. System Organ Class (SOC) –showed that there is no clear relationship between the number of SIDER-only associations in a category and the change in AUROC when SIDER is used for validation instead of Lexi-comp (Table 3). In fact, the relative change in AUROC was less than 5% for all but six SOCs which are rather general and not specifically related to a body organ or system: 1) Congenital, familial and genetic disorders (relative change 34%), 2) Surgical and medical procedures (relative change 20%), 3) General disorders (relative change 12%), 4) Injury, poisoning and procedural disorders (relative change 7%), 5) Investigations (relative change 7%), 6) Pregnancy, puerperium and perinatal conditions (relative change 5.1%).

**Table 3:** AUROC of PPN by MedDRA top-level category

**MedDRA top-level category**	**Lexi-comp 2010**	**SIDER 2012**
	**AUROC (95% CI)**	**AUROC (95% CI)**
Nervous system disorders	0.85 (0.84 – 0.86)	0.84 (0.84 – 0.85)
Gastrointestinal disorders	0.85 (0.84 – 0.86)	0.84 (0.83 – 0.85)
Skin and subcutaneous tissue disorders	0.85 (0.84 – 0.86)	0.85 (0.85 – 0.86)
**General disorders**	**0.86 (0.84 – 0.87)**	**0.76 (0.75 – 0.77)**
Respiratory, thoracic and mediastinal disorders	0.80 (0.79 – 0.82)	0.78 (0.77 – 0.79)
Vascular disorders	0.84 (0.83 – 0.86)	0.83 (0.82 – 0.84)
Psychiatric disorders	0.83 (0.82 – 0.85)	0.81 (0.81 – 0.82)
Musculoskeletal and connective tissue disorders	0.82 (0.80 – 0.84)	0.83 (0.82 – 0.84)
Eye disorders	0.79 (0.77 – 0.81)	0.83 (0.82 – 0.84)
Infections and infestations	0.85 (0.83 – 0.86)	0.85 (0.84 – 0.86)
**Investigations**	**0.86 (0.84 – 0.88)**	**0.80 (0.79 – 0.81)**
Metabolism and nutrition disorders	0.81 (0.79 – 0.83)	0.82 (0.81 – 0.83)
Blood and lymphatic system disorders	0.87 (0.86 - 0.89)	0.88 (0.87 – 0.88)
Cardiac disorders	0.83 (0.81 – 0.85)	0.84 (0.82 – 0.85)
Renal and urinary disorders	0.86 (0.84 – 0.87)	0.85 (0.83 – 0.86)
Reproductive system and breast disorders	0.85 (0.82 – 0.87)	0.86 (0.84 – 0.87)
Immune system disorders	0.88 (0.87 – 0.90)	0.90 (0.89 – 0.91)
Neoplasms benign, malignant and unspecified	0.80 (0.74 – 0.85)	0.78 (0.76 – 0.80)
Hepatobiliary disorders	0.85 (0.83 – 0.88)	0.83 (0.81 – 0.84)
**Injury, poisoning and procedural disorders**	**0.86 (0.84 – 0.89)**	**0.80 (0.78 – 0.82)**
Endocrine disorders	0.82 (0.79 – 0.85)	0.85 (0.83 – 0.86)
Ear and labyrinth disorders	0.86 (0.83 – 0.89)	0.84 (0.82 – 0.86)
**Surgical and medical procedures**	**0.82 (0.73 – 0.89)**	**0.66 (0.60 – 0.72)**
**Pregnancy, puerperium and perinatal conditions**	**0.78 (0.63 – 0.93)**	**0.74 (0.66 – 0.82)**
**Congenital, familial and genetic disorders**	**0.91 (0.83 – 0.98)**	**0.60 (0.53 – 0.66)**

Note: Rows are shown in decreasing order of the number of SIDER-only associations. Bold-face: Six MedDRA categories with the highest AUROC difference between SIDER 2012 and Lexi-comp 2010.

## Discussion

This study aimed to systematically assess the concordance between Lexi-comp and SIDER ADE data sets, as well as the impact of using each data set in the prediction performance of PPN model. Our main result was that ADE and drug counts in the Lexi-comp 2010 and SIDER 2012 data sets were highly correlated and that the AUROC of the PPN model changed very little (approximately 1.1%) when SIDER 2012 was used for validation instead of Lexi-comp 2010.

While we found overall concordance, there were also differences between the Lexi-comp 2010 and SIDER 2012 data sets. These differences were most pronounced for Nervous System and Anti-infective drugs, for Gastrointestinal and Nervous System ADEs, and for “postmarketing” ADEs. Our results suggest that the differences between the two data sets do not simply arise from the two-year time lag between them. Indeed, the correlations of drug and ADE counts were higher between the two Lexi-comp snapshots (separated by five years) than they were between Lexi-comp 2010 and SIDER 2012. Further, relative to Lexi-comp 2005, SIDER 2012 introduces new associations at a higher rate than Lexi-comp 2010. As discussed earlier, other factors that could have introduced these differences include the use of various sources to supplement package-insert information and the independent mapping of ADE names to MEdDRA.

The observations of high overall concordance between Lexi-comp and SIDER, and high robustness of the PPN model under two different validation sets, are not affected by the time lag between the two data sets. In fact, the presence of the time lag makes the concordance and robustness conclusions even stronger than they would be if the same results were obtained by comparing data from the same year. On the other hand, the differences between the two data sets should be interpreted with caution as it is not clear to what extent they are accounted for by the time lag and to what extent by other factors. The interpretation of these differences is also hindered by the high proportion of missing ADE frequency data (63%).

While this manuscript was under preparation, Lin et al. [27] published a new study in which they developed an “external link prediction” method for unknown drug-ADE associations. Using two snapshots of data based on the intersection of SIDER with FAERS 2005 and FAERS 2011, respectively, they carried out a simulated prospective validation of a subset of PPN covariates analogous to the validation by Cami et al. [21]. The training set in the study by Lin et al. consisted of 422 drugs and 462 ADEs. These authors found that in that data set, the chosen subset of PPN covariates achieved an AUROC of 0.75, while the “external link prediction” method achieved an AUROC of 0.83. Thus, the study by Lin et al. using a different ADE data source, different validation year and different drug and ADE sets provides an independent confirmation of the robustness of PPN variables with respect to the choice of ADE data set.

Recently, Tatonetti et al. [24] published a method to extract potentially significant drug-ADE associations from FAERS and a new accompanying data set of such associations (OFFSIDES). Similarly, Cheng et al. [25] developed a new drug-ADE data set named MetaADEDB by integrating information from SIDER, CTD (ctdbase.org), and OFFSIDES, and utilizing Medical Subject Headings (MeSH) to annotate compounds and diseases. We believe that these data integration, standardization and annotation efforts are important steps toward the development of improved reference standards for drug-ADE associations.

## Conclusions

In summary, we have conducted a study that systematically compared two drug safety data sets and assessed the impact of data set choice on the prediction performance of the PPN predictive model. Overall, we found a high concordance between the two data sets and only a minor impact on the prediction performance of PPN. However, we also identified a number of key differences between the two data sets. We believe it is important for researchers, drug safety professionals and public health officials to be aware of such differences, especially in modeling applications aimed at specific drug and ADE categories, and a wide range of studies aimed at ADE prediction models.

## Competing interests

The authors declare that they have no competing interests.

## Authors’ contributions

AC and BR acquired the data, developed the proposed methods and designed the study. AC implemented the methods and performed the experiments. AC and BR wrote the manuscript. Both authors read and approved the final manuscript.

## Pre-publication history

The pre-publication history for this paper can be accessed here:

http://www.biomedcentral.com/1472-6947/14/74/prepub
